# *Gastrodia elata* Polysaccharide Attenuates Aging by Modulating Oxidative Stress, Inflammation, and Gut Microbiota

**DOI:** 10.1002/fsn3.72413

**Published:** 2026-09-30

**Authors:** Huihuang Shi, Shunqiang Yang, Yue Chen, Biting Shaog, Rui Gong, Zhu Li

**Affiliations:** ^1^ Yunnan Key Laboratory of Gastrodia and Fungi Symbiotic Biology College of Agronomy and Life Sciences, Zhaotong University Zhaotong China; ^2^ Yunnan Engineering Research Center of Green Planting and Processing of Gastrodia College of Agronomy and Life Sciences, Zhaotong University Zhaotong China; ^3^ Yunnan Key Laboratory of Smart Villages and Agri‐Cultural‐Tourism Integration Zhaotong University Zhaotong China

**Keywords:** aging, *Gastrodia elata* polysaccharide (GEP), gut microbiota, inflammation, oxidative stress

## Abstract

Aging is a natural, progressive physiological process accompanied by systemic multi‐organ dysfunction, for which effective intervention strategies remain limited. *Gastrodia elata* is a well‐known food and medicine homologous resource with extensive bioactive properties. *Gastrodia elata* polysaccharide (GEP), a bioactive macromolecule isolated from *G. elata*, is a promising candidate for the development of anti‐aging functional products. In this study, we first verified the preliminary anti‐aging effects of GEP in vitro and in *Caenorhabditis elegans* , and further administered GEP to natural aging mice to explore its anti‐aging effects and underlying mechanisms. The results showed that GEP significantly alleviated d‐galactose (d‐gal)‐induced senescence of BV2 microglia, prolonged the lifespan of nematodes, and in aging mice, it markedly ameliorated cognitive impairment, muscle dysfunction, and hippocampal neuronal damage, while upregulating the expression of neuroprotective BDNF. Moreover, GEP reduced the level of malondialdehyde (MDA) and increased the activities of superoxide dismutase (SOD) and catalase (CAT), and activated the Nrf2/Keap1 signaling pathway by downregulating Keap1 and upregulating HO‐1 and NQO1, suggesting that GEP may ameliorate aging by reversing oxidative stress imbalance. GEP also significantly decreased the levels of pro‐inflammatory factors tumor necrosis factor‐α (TNF‐α), interleukin‐1β (IL‐1β), and interleukin‐6 (IL‐6) in both serum and brain tissue, which may be the result of the alleviation of chronic inflammation by the inactivation of the TLR4/NF‐κB pathway. Furthermore, our results revealed a significant association between GEP‐mediated gut microbial remodeling and its neuroprotective phenotypes. GEP normalized aging‐induced gut dysbiosis, recovered microbial diversity, restored the abundance of beneficial genera including *Akkermansia* and *Lactobacillus*, and elevated intestinal tight junction proteins ZO‐1 and occludin. Such intestinal improvements may partially contribute to GEP's anti‐aging capacity through the gut–brain axis, though definitive causal evidence such as fecal microbiota transplantation is lacking in the present study. Collectively, these findings provide a theoretical foundation for the development and application of GEP‐based functional products targeting aging intervention.

## Graphical abstract

*Gastrodia elata* polysaccharide (GEP) alleviates aging in mice by activating the Nrf2/Keap1 antioxidant signaling, inhibiting the TLR4/NF‐κB‐driven neuroinflammation, remodeling gut microbiota, and restoring intestinal‐barrier integrity. Synergistic regulation of oxidation, inflammation, and gut homeostasis underpins its anti‐aging potential for functional‐food applications.

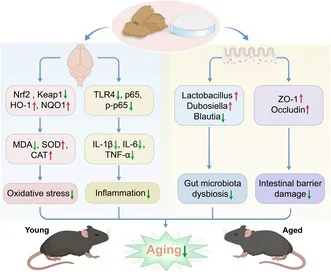

## 1 Introduction

Aging is an irreversible process characterized by structural damage and functional decline of cells, tissues, and organs induced by advancing age (He and Sharpless 2017), which is jointly regulated by multiple factors including genetics, environment, and diet, and modulated by interconnected molecular and metabolic pathways (Kennedy et al. 2014; Widjaja et al. 2024; Argentieri et al. 2025). At present, the global aging trend is intensifying, and aging‐related diseases such as cardiovascular diseases, metabolic disorders, kidney diseases, and neurodegenerative diseases are prevalent, which seriously reduce the quality of life of the elderly and impose a heavy social and economic burden (Ye et al. 2023; Schmanske et al. 2025; Sun and Chen 2025). Therefore, it is of great social and economic value to deeply analyze the mechanisms of aging, explore active substances that delay aging, and reduce the incidence of aging‐related diseases.

Compared with single pathological intervention, systemic regulation of aging is considered a more effective strategy to improve the health of the elderly and prolong healthspan (Garmany et al. 2021). Cellular senescence is the core hallmark and main driving force of aging (López‐Otín et al. 2023; Sun and Chen 2025; Calcinotto et al. 2019; Suryadevara et al. 2026). The continuous accumulation of senescent cells triggers pathological changes in tissues and accelerates organ degeneration, making it an important target for anti‐aging research (Pignolo et al. 2020; Xu et al. 2021; Baier et al. 2025). Oxidative stress is a key inducer accelerating cellular senescence (Tao et al. 2024; Wang, Ge, et al. 2025). During aging, the disorder of oxidative stress response and antioxidant repair capacity directly leads to cellular senescence and death (Hauser et al. 2015; Snieckute et al. 2023). Meanwhile, oxidative stress activates inflammatory signals and promotes the release of inflammatory factors; excessive inflammatory factors in turn disrupt cellular metabolism and mitochondrial function, aggravate reactive oxygen species (ROS) production, forming a vicious cycle between oxidative stress and inflammation. The two factors jointly drive the progression of aging and related diseases (Trushina and McMurray 2007; Guzik and Touyz 2017; Kumar et al. 2023). In addition, the intestine, with its immune and nutrient absorption functions, is a key organ regulating organismal lifespan (Choi and Augenlicht 2024). Metchnikoff proposed that “aging begins in the gut,” arguing that toxins produced by gut microbiota are important triggers of organismal aging and diseases (Cavaillon and Legout 2016). Gut microbiota, known as the “hidden organ” (de Vos et al. 2022), undergoes dysregulation during aging, manifested as decreased beneficial bacteria, elevated lipopolysaccharide and inflammatory levels, and impaired intestinal barrier, which further accelerate aging (Bana and Cabreiro 2019; Ticinesi et al. 2026). Studies have confirmed that regulating microbial structure and restoring beneficial bacteria levels can improve intestinal barrier function and delay intestinal aging (Ma et al. 2023). Gut microbiota acts as a core regulator of the aging trajectory, and shapes health‐ or disease‐associated aging phenotypes via three major pathways: the gut–multi‐organ axis, inflammaging, and cellular senescence (Ticinesi et al. 2026). Therefore, oxidative stress, inflammatory response, and gut microbiota can all be used as evaluation indicators of aging.

Although aging is irreversible, its progression can be delayed by rational interventions. Traditional Chinese medicine (TCM) has unique theoretical and practical advantages in the field of anti‐aging. With the characteristics of multi‐component, multi‐target, low toxicity, and side effects, TCM fits the complex mechanism of aging and has become a research hotspot (Zhuang et al. 2026). *Gastrodia elata* is a classic traditional Chinese herb with dual medicinal and edible properties, clinically used to manage epilepsy, dizziness, limb numbness, hypertension, and pediatric convulsions (Matias et al. 2016; Li et al. 2025). Modern studies have shown that it exerts neuroprotective, memory‐improving, antioxidant, anti‐brain aging, and other diverse activities (Gong et al. 2024; Wang, Hu, et al. 2025; Zhou et al. 2024; Liu et al. 2025). Polysaccharides, with high safety, are the focus of health food and healthcare product development (Liu et al. 2023; Xu et al. 2026; Liao et al. 2026). *Gastrodia elata* polysaccharide (GEP) is the most abundant component in *G. elata*. As a novel bioactive macromolecule, GEP has been proven to exert multi‐dimensional pharmacological effects, including anti‐inflammation, anti‐apoptosis, inhibition of oxidative stress, immune enhancement, blood pressure and lipid regulation, and neuroprotection (Dai et al. 2021; Wang, Qi, et al. 2025; Zhu et al. 2024). In addition, studies have shown that GEP can regulate the composition of gut microbiota (Gan et al. 2024; Chen, Li, et al. 2025). However, it remains unclear whether GEP exerts neuroprotective effects by modulating gut microbiota and serves as a candidate agent for alleviating aging.

In summary, inhibiting oxidative stress and inflammation, regulating gut microbiota, and repairing intestinal mucosal barrier are important approaches to delay aging. In the present study, we found that GEP alleviated d‐galactose (d‐gal)‐induced senescence of BV2 microglia and prolonged the lifespan of *Caenorhabditis elegans* N2, and its efficacy was further verified in naturally aging mice. We demonstrated that GEP exerts anti‐aging effects by inhibiting oxidative stress, alleviating inflammatory response, regulating gut microbiota, and improving intestinal barrier function. This study provides a theoretical basis for the research and application of GEP‐based functional products from the dual dimensions of neuroprotection and gut microbiota regulation, and offers a new direction for the research of natural anti‐aging substances.

## 2 Materials and Methods

### 2.1 Preparation of GEP

*Gastrodia elata* was collected from Xiaocaoba, Zhaotong City, Yunnan Province, China. GEP was extracted with reference to previous literature (Chen et al. 2018). Briefly, 200 g of dried *G. elata* powder was added to 3 L of distilled water and extracted at 100°C for 4 h to obtain the aqueous extract. After cooling to room temperature, the extract was centrifuged at 6000 r/min for 20 min and the supernatant was collected and concentrated. Subsequently, four volumes of ethanol were added to the supernatant to achieve a final ethanol concentration of no less than 80%, and the mixture was allowed to stand overnight before centrifugation. The precipitate was collected and redissolved in H_2_O. After deproteinization by the Sevag method, the solution was freeze‐dried to obtain GEP. The total sugar content of GEP was determined by the phenol–sulfuric acid method, and the protein content was detected using a BCA kit.

### 2.2 Characteristics of GEP

#### 2.2.1 Molecular Weight of GEP

The molecular weight of GEP was determined by high‐performance gel permeation chromatography (HPGPC) with reference to and optimization of previous methods (Gan et al. 2024). Briefly, GEP was prepared into a 10 mg/mL solution, centrifuged at 6000 r/min for 10 min, filtered through a 0.22 μm membrane, and the filtrate was collected for injection analysis. Chromatographic detection was performed using an Agilent 1100 Series high‐performance liquid chromatography system (Agilent Technologies, USA) equipped with a refractive index detector (RID) for signal acquisition; a TSK‐gel G3000 PWXL column (7 μm, 7.8 × 300 mm, TOSOH, Japan) was used. Chromatographic conditions: mobile phase was 0.05 mol/L sodium sulfate solution, flow rate 0.5 mL/min, column temperature 40°C, injection volume 20 μL. A series of dextrans with different molecular weights provided by the National Institutes for Food and Drug Control were used as standards to prepare standard solutions of the same concentration. A standard curve was constructed by plotting the logarithm of molecular weight (log Mw) against retention time, establishing the linear regression equation: *y* = −2.4034*x* + 23.635 (*R* ^2^ = 0.9906), with a linear range of 180–300,600 Da.

#### 2.2.2 Monosaccharide Composition of GEP

The monosaccharide composition of GEP was determined by 1‐phenyl‐3‐methyl‐5‐pyrazolone (PMP) pre‐column derivatization‐high‐performance liquid chromatography (HPLC) with reference to and optimization of existing methods (Gan et al. 2024). Briefly, 5 mg of GEP sample was placed in an ampoule, dissolved thoroughly with 1 mL of ultrapure water, and 1 mL of 4 M trifluoroacetic acid (TFA) solution was added. After sealing the ampoule with a spray gun, it was heated in an oven at 110°C for 4 h for hydrolysis. After hydrolysis, the sample solution was neutralized to neutrality with sodium hydroxide (NaOH) solution. 200 μL of the neutralized sample solution was mixed with 200 μL of internal standard solution, 100 μL of which was transferred to a centrifuge tube. Then, 100 μL of 0.3 mol/L NaOH solution and 0.5 mol/L PMP derivatization solution were added successively, and derivatization was carried out in a water bath at 70°C in the dark for 1 h. After cooling, the solution was neutralized with 0.3 mol/L hydrochloric acid. 500 μL of chloroform was added to the derivatized solution for extraction, centrifuged at 7000 rpm for 5 min, and the upper aqueous phase was collected. The extraction was repeated four times to remove residual reagents and impurities, and the aqueous phase was filtered through a 0.22 μm aqueous membrane for testing. Chromatographic detection was performed using a Shimadzu LC‐2030C 3D Plus high‐performance liquid chromatograph with a Shim‐pack GIST column (5 μm, 4.6 × 150 mm); detection wavelength 254 nm, column temperature 30°C, flow rate 1 mL/min; mobile phase A was 83% phosphate buffer, mobile phase B was 17% acetonitrile, with gradient elution for separation. Thirteen monosaccharide standards including mannose, rhamnose, and glucose were used as controls. The monosaccharide components of GEP were qualitatively identified based on retention time, and quantitative analysis of monosaccharide composition was completed by the peak area method.

#### 2.2.3 Fourier Transform Infrared Spectroscopy of GEP

Fourier transform infrared spectroscopy (FT‐IR) was used to characterize the functional groups and structural characteristics of GEP with reference to and optimization of existing methods (Gan et al. 2024). A Nicolet iS5 Fourier transform infrared spectrometer (Nicolet iS5, Thermo Fisher) was used for the experiment. 1 mg of dried GEP sample was mixed with 49 mg of potassium bromide (KBr) powder at a mass ratio of approximately 1:49 (sample accounting for about 2%), ground thoroughly in an agate mortar under an infrared lamp, loaded into a tablet mold, and pressed into a uniform and transparent KBr sample tablet by applying a pressure of 10 T for 30 s using a tablet press. After preheating and stabilizing the instrument, the blank KBr background spectrum was collected first, then the sample tablet was placed in the optical path, and scanning was performed in the wavenumber range of 4000–400 cm^−1^ to obtain the infrared absorption spectrum. Background subtraction and baseline correction were performed on the original spectrum, and the main functional groups and sugar chain structural characteristics of GEP were analyzed according to the wavenumber positions of characteristic absorption peaks.

### 2.3 Cell Culture and Treatments

BV2 microglial cell lines were maintained under standard culture conditions as detailed in our earlier work (Song et al. 2014). In brief, cells were grown in high‐glucose Dulbecco's modified Eagle's medium (DMEM) supplemented with 10% (v/v) fetal bovine serum and incubated in a humidified atmosphere with 5% CO_2_ at 37°C. All experiments were conducted when cells reached the logarithmic growth phase. To induce cellular senescence in vitro, BV2 cells were exposed to a series of d‐gal concentrations (0, 50, 100, 200, and 400 mM) for 24 h. For the intervention assay, cells were co‐treated with 200 mM d‐gal and graded concentrations of GEP (20, 40, and 80 μg/mL) for the same incubation period.

### 2.4 Cell Viability Assay Using CCK‐8

Cell viability was measured using a Super‐Enhanced Cell Counting Kit‐8 (CCK‐8, Beyotime, C0048) referring to our previous study (Li et al. 2026). Briefly, cells in the logarithmic growth phase were digested with 0.25% trypsin, and resuspended in complete culture medium to generate a single‐cell suspension at a density of 5 × 10^4^ cells/mL. Each well of a 96‐well plate was seeded with 100 μL of the cell suspension and pre‐incubated for 20 h at 37°C with 5% CO_2_ to ensure full cell adherence. Then, culture media containing different concentrations of test compounds were added to each well, with three technical replicates set for each treatment group. After 24 h of incubation, 10 μL of CCK‐8 reagent was added to each well, followed by an additional 1 h incubation in the dark. The absorbance at 450 nm was measured using a microplate reader to reflect relative cell viability.

### 2.5 β‐Galactosidase (SA‐β‐Gal) Staining

Senescence‐associated β‐galactosidase (SA‐β‐gal) activity was detected using a commercial staining kit (Cell Signaling, 9860) with minor modifications to the manufacturer's protocols (Hsu et al. 2021; Kang et al. 2024). Briefly, BV2 cells were seeded onto glass coverslips placed in 12‐well plates and cultured for 24 h prior to drug treatment. After the designated treatment, the culture medium was carefully removed, and cells were rinsed once with 1 mL of pre‐cooled 1× phosphate‐buffered saline (PBS). Then, 400 μL of SA‐β‐gal fixative solution was added to each well for 15 min fixation at ambient temperature. After removing the fixative, cells were washed three times with 1× PBS (3 min per wash). Following complete removal of PBS, 400 μL of freshly prepared SA‐β‐gal staining working solution was added to each well. The plates were sealed and incubated overnight at 37°C without CO_2_, and positive staining was observed and photographed under an optical microscope. For quantitative detection of SA‐β‐gal activity, a 96‐well Cellular Senescence Assay Kit (Cell Biolabs, CBA‐231) was applied according to the manufacturer's instructions. Fluorescence intensity was measured at excitation 360 nm and emission 465 nm with a fluorescence microplate reader.

### 2.6 Effect of GEP on Nematode Lifespan

The life cycle of *C. elegans* is divided into embryonic stage, four larval stages (L1–L4), and adult stage (Marchoom‐Bura et al. 2025). In this study, *C. elegans* were treated at the L1 stage. Synchronized L1‐stage N2 nematodes were transferred to NGM solid medium plates containing gradient doses of GEP (0.25, 0.5, 1.0 mg/mL), and nematodes cultured on NGM plates without GEP were used as the blank control (Control), with 50 nematodes per group. The nematodes were incubated at 20°C and transferred to fresh NGM plates daily to maintain a constant GEP concentration and avoid interference from nematode offspring. Given that many plant polysaccharides possess antibacterial properties (Zhou et al. 2023; Ma, Jiang, et al. 2026), which may indirectly induce caloric restriction (a well‐recognized lifespan‐extending intervention) by inhibiting bacterial growth, heat‐inactivated *Escherichia coli* OP50 (inactivated at 65°C for 0.5 h) was used as the uniform food source for all groups to exclude this potential confounding effect. The number of surviving nematodes was recorded daily. Nematodes were considered dead if they did not respond to gentle touch on the head or tail with a platinum wire. Nematodes that died of dehydration on the plate wall or exhibited internal hatching (“bagging”) were treated as right‐censored data in survival analysis: they were excluded from death event statistics but retained in the dataset up to their censoring time. Survival curves were plotted using the Kaplan–Meier method, and intergroup differences in survival rates were analyzed using the log‐rank test. Lifespan assays were performed in at least three independent experiments.

### 2.7 Animals

Male C57BL/6J mice (8 weeks old, weighing 25 ± 2 g) were purchased from Jiangsu Jicui Yaokang Biotechnology Co. Ltd. All mice were housed in a specific pathogen‐free (SPF) animal facility under controlled conditions: 12‐h light/dark cycle (7:00 a.m. to 7:00 p.m. light phase), 55% relative humidity, and a temperature of 22°C ± 1°C, with free access to standard laboratory chow and sterile water. All mice were of the same strain, sex, and genetic background to minimize confounding factors. All experimental protocols were reviewed and approved by the Animal Ethics Committee of Zhaotong University (approval no. 20230003A9) to ensure strict adherence to ethical standards. This study complied with the guidelines of the National Institutes of Health (NIH) Guide for the Care and Use of Laboratory Animals, an internationally recognized framework for the humane treatment of research animals. Furthermore, all experimenters held valid certifications for laboratory animal manipulation.

### 2.8 Animal Grouping and Experimental Administration

A natural aging model was adopted in this study. Fifteen 2‐month‐old young mice were used as the normal young group (Young). Seventy‐five 18‐month‐old male C57BL/6J mice were randomly divided into five groups according to body weight: natural aging model group (Aging), aging + vitamin E group (100 mg/kg, Aging_VitE), aging + low‐dose GEP group (100 mg/kg, Aging_GEP_L), aging + medium‐dose GEP group (200 mg/kg, Aging_GEP_M), and aging + high‐dose GEP group (400 mg/kg, Aging_GEP_H). Mice were intragastrically administered (i.g.) for two consecutive months. The mental state, diet, skin, and hair of mice were observed during administration. One week of adaptive feeding was conducted before formal experiments after administration.

### 2.9 Behavioral Tests

Behavioral tests were performed 1 week after the end of administration.

#### 2.9.1 Morris Water Maze Tests

Spatial cognitive function of mice was evaluated using the Morris water maze (MWM) test with minor modifications based on our previous studies (Li et al. 2020; Chen, Yang, et al. 2025). During place navigation training, the platform was submerged 0.5–1 cm beneath the water surface and rendered invisible with white dye. Mice were released from four quadrants facing the inner pool wall, and their swimming trajectories and escape latency were recorded by a video tracking system. Mice failing to locate the platform within 90 s were given a maximum latency of 90 s, then guided to the platform for a 30‐s stay to reinforce spatial memory. Following the 5‐day place‐navigation training regimen, the spatial probe trial was conducted on Day 7. The hidden platform was removed from the circular pool (1 m diameter, 35 cm height), and mice were released into the water from different quadrants to swim freely for 90 s. With fixed visual cues of various colors and shapes on the pool walls as spatial references, the spatial memory of mice was assessed by recording three key indicators, including swimming trajectories, the total time spent in the target quadrant, and the number of crossings over the original platform position in the training stage.

#### 2.9.2 Nest Building Tests

The nest building test was performed with minor modifications based on previous studies (Deacon 2006; Hsu et al. 2021). One hour before the dark cycle, nesting materials, food, and water were provided ad libitum in mouse cages. After overnight nesting, the nests were blindly scored by two independent observers using the Nesting Score System (NSS, 0–5): 0 = no material use; 1 = disturbed but unshaped material; 2 = flat dish‐like nest with incomplete walls; 3 = cup‐shaped nest with clear walls; 4 = incomplete dome nest; 5 = complete dome nest with one small entrance. A quiet, undisturbed environment was maintained throughout the experiment.

#### 2.9.3 Grip Strength Test and Endurance Test

Muscle strength and function decline gradually with aging. Forelimb grip strength and wire mesh climbing endurance tests were used to evaluate muscle function in mice according to a previous study (Xie 2024). In the grip strength test, mice were placed on the grip meter bar and held steadily with four limbs; their tails were gently pulled backward horizontally, and the grip value was recorded. Each mouse was tested five times, with the maximum and minimum values excluded; the remaining highest value was used for analysis after body weight correction. Mice that failed to grasp firmly or struggled were retested after a rest. For the endurance test, a circular wire mesh (diameter 600 mm) was fixed 60 mm above the water surface. The duration from placement to falling into the water due to exhaustion was recorded as climbing time. Ten mice per group (excluding the heaviest and lightest) were tested three times with 3‐min intervals; the longest time was collected and corrected by body weight to represent endurance.

### 2.10 Sample Preparation

After behavioral tests, mice were fasted for 12 h with free access to water, and feces were collected and stored at −80°C. The next day, mice were deeply anesthetized with carbon dioxide. After complete anesthesia, blood was collected from the eyeballs. Whole blood samples were centrifuged at 3000 r/min for 20 min at 4°C, and the supernatant (serum) was collected and stored at −80°C for later use. After blood collection, some mice were perfused transcardially with 20 mL of pre‐cooled 1× PBS (pH 7.4) buffer until the liver turned pale yellow. Brain and colon tissues were rapidly dissected and fixed in 4% paraformaldehyde for 48 h for subsequent histological analysis. The remaining brain and colon tissues were stored at −80°C for later use.

### 2.11 Hematoxylin–Eosin (H&E) Staining, Immunohistochemical (IHC) Staining, and Immunofluorescence (IF) Staining

H&E staining, IHC staining, and IF staining were performed with minor modifications based on our previous protocol (Li et al. 2024, 2026; Chen, Yang, et al. 2025). Briefly, brain tissues were fixed in 4% paraformaldehyde, paraffin‐embedded, and cut into 5‐μm sections. H&E staining was conducted on dewaxed and dehydrated paraffin sections following standard procedures. IHC staining was used to detect BDNF (1:100, GB11559, Servicebio) expression in brain tissues. For IF staining, colon tissues were frozen and sectioned into 20‐μm coronal slices. After rinsing, sections were permeabilized with 0.1% Triton X‐100 for 20 min, blocked with 1.5% fetal bovine serum for 1 h at room temperature, then incubated with primary antibodies against ZO‐1 (1:1000, GB151981, Servicebio) and Occludin (1:1000, GB111401, Servicebio) overnight at 4°C. Following PBS washing, sections were incubated with fluorescence‐conjugated secondary antibodies. IF images were acquired using a confocal laser scanning microscope (LSM980, Carl Zeiss).

### 2.12 Determination of Inflammatory and Oxidative Stress Factors

The content of malondialdehyde (MDA, Solarbio, BC6415) and the activities of superoxide dismutase (SOD, Solarbio, BC0175) and catalase (CAT, Solarbio, BC4785) were determined using commercial kits. In addition, the levels of pro‐inflammatory cytokines including interleukin‐1β (IL‐1β, Solarbio, CEA‐C250), interleukin‐6 (IL‐6, Solarbio, CEA‐M254), and tumor necrosis factor‐α (TNF‐α, Solarbio, CEA‐M255) in serum and brain tissue were quantified via commercial enzyme‐linked immunosorbent assay (ELISA) kits.

### 2.13 Western Blot

Protein samples were denatured in 1 × SDS loading buffer, separated by 10% SDS‐PAGE, and transferred onto PVDF membranes. Membranes were incubated with primary antibodies against Nrf2 (1:400, sc‐365949, Santa Cruz), Keap1 (1:800, A25951, ABclonal), HO‐1 (1:500, ab13248, Abcam), NQO1 (1:5000, A19586, ABclonal), TLR4 (1:600, Sc‐293072; Santa Cruz), P65 (1:600, Sc‐8008; Santa Cruz), phospho‐P65 (1:600, Sc‐136548, Santa Cruz), and GAPDH (1:10,000, 60004‐1‐Ig, Proteintech). After washing, membranes were incubated with HRP‐conjugated secondary antibody (1:5000, A0545, Sigma). Protein bands were visualized by enhanced chemiluminescence (ECL) and quantified using ImageJ software (NIH).

### 2.14 Gut Microbiota Analysis

Mouse fecal samples were sent to Novogene Co. Ltd. (Beijing) for 16S rRNA sequencing and species identification. Briefly, the process included genomic DNA extraction and PCR amplification, pooling, and purification of PCR products, library construction and sequencing, and finally bioinformatic analysis.

### 2.15 Statistical Analysis

Histological staining (H&E, IHC, IF) and behavioral scoring (nest building test) were blindly assessed by two independent observers to avoid subjective bias. One‐way ANOVA was used for comparisons among multiple groups. When a significant main effect was observed (*p* < 0.05), Dunnett's multiple comparisons test was adopted with the Aging group as the reference for pairwise comparisons. Statistical analyses were conducted with SPSS 22.0 and Prism 9.0 software. All data are presented as mean ± standard deviation (SD), and *p* < 0.05 was regarded as statistically significant.

## 3 Results

### 3.1 Structural Characterization of GEP

To characterize the structural properties of purified GEP, we first performed a series of physicochemical analyses. The results showed that the total sugar content of GEP was 90.04%, and the protein content was only 1.72%, indicating a high‐purity polysaccharide component. HPGPC analysis showed that GEP presented a single symmetric elution peak at 9–12 min. Calculated by the dextran standard curve, its average molecular weight was 31.48 × 10^4^ Da (Figure 1A). Monosaccharide composition analysis showed that GEP was mainly composed of glucose (Figure 1B). FT‐IR results further verified the sugar structural characteristics of GEP: the strong absorption peak at 3415.65 cm^−1^ was the stretching vibration of polysaccharide —OH, indicating the presence of intermolecular and intramolecular hydrogen bonds in GEP (Gan et al. 2022). The characteristic absorption peak at 2928.89 cm^−1^ was attributed to the C—H stretching vibration of —CH_2_— or —CH—, the peak at 1636.25 cm^−1^ corresponded to the asymmetric stretching vibration of C=O in —COOH, the peak at 1368.44 cm^−1^ was assigned to the C—H bending vibration of —CH_2_— or —CH—, the multiple peaks at 1054.11, 1080.39, and 1022.87 cm^−1^ belonged to the C—O—C and C—O—H stretching vibrations of the pyranose ring, and the peak at 925.50 cm^−1^ was attributed to the asymmetric stretching vibration of C—O—C in d‐glucopyranose (Wang et al. 2021; Teng et al. 2021; Zhang et al. 2023). The absorption peak at 851.41 cm^−1^ indicated the presence of α‐glycosidic bonds and the absorption peak at 762.24 cm^−1^ corresponded to the characteristic absorption peak of α‐d‐pyranose (Yang et al. 2021; Chen et al. 2019) (Figure 1C). These results collectively indicated that GEP was mainly a macromolecular polysaccharide with an α‐glucopyranose structure and a molecular weight of 31.48 × 10^4^ Da.

**FIGURE 1:**
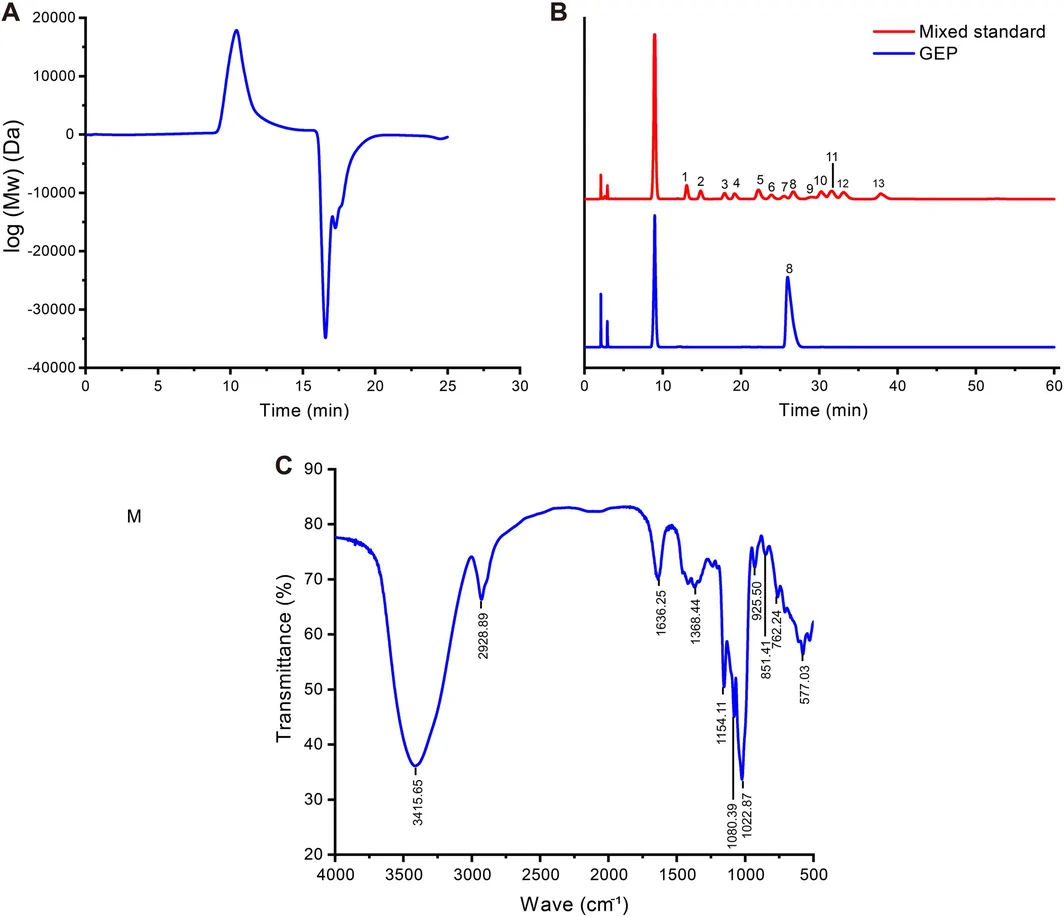
Structural characteristics of GEP. (A) Determination results of the molecular weight of GEP. (B) Monosaccharide composition analysis of GEP: 1. Mannose; 2. Glucosamine; 3. Rhamnose; 4. Glucuronic acid; 5. Galacturonic acid; 6. Galactosamine; 7. N‐acetylglucosamine; 8. Glucose; 9. N‐acetylgalactosamine; 10. Galactose; 11. Xylose; 12. Arabinose; 13. Fucose. (C) Fourier transform infrared spectroscopy of GEP.

### 3.2 GEP Alleviates d‐Gal‐Induced Senescence of BV2 Cells and Prolongs Lifespan of N2 Nematodes

To preliminarily verify the anti‐aging activity of GEP, we first established a d‐gal‐induced senescence model in BV2 microglia. CCK‐8 assay showed that after treatment of BV2 cells with different concentrations of d‐gal for 24 h, cell viability decreased significantly in a concentration‐dependent manner. Among them, 200 mM d‐gal reduced cell viability to approximately 50%, which was a suitable modeling concentration (Figure 2A). SA‐β‐gal staining further confirmed that after treatment with 200 mM d‐gal, the positive rate of senescent cells was significantly higher than that in the control group, indicating successful establishment of the senescence model (Figure 2B). On this basis, we co‐treated cells with different concentrations of GEP and d‐gal. The results showed that GEP reversed d‐gal‐induced decrease in cell viability in a concentration‐dependent manner, and significantly reduced the proportion of SA‐β‐gal positive senescent cells. Among them, 40 μg/mL GEP showed the most significant ameliorative effect (Figure 2C). In addition, the survival curve of N2 nematodes showed that GEP intervention significantly prolonged the average lifespan of nematodes, and 0.5 mg/mL GEP showed the best effect (Figure 2D).

**FIGURE 2:**
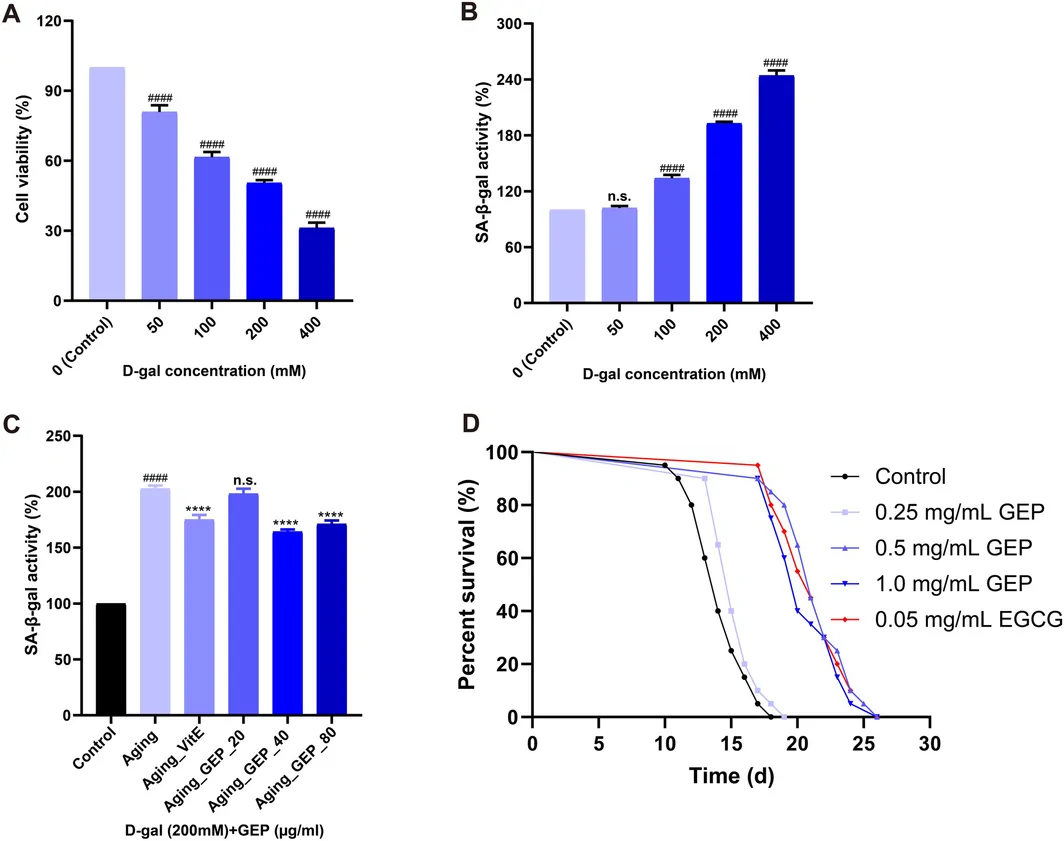
GEP reduces d‐gal‐induced cellular senescence and prolongs the lifespan of N2 nematodes. (A) CCK‐8 assay of BV2 cells treated with d‐gal (0–400 mM) for 24 h. (B) SA‐β‐gal staining of BV2 cells treated with d‐gal (0, 50, 100, 200, and 400 mM) for 24 h. (C) BV2 cells were co‐treated with GEP (0, 5, 10, 20, 40, and 80 μg/mL) and 200 mM d‐gal for 24 h. (D) Survival curve of N2 nematodes. All experimental data are presented as mean ± SD. *n* = 6, ns > 0.05, ^####^ *p* < 0.0001 versus Control group; ****p < 0.0001 versus d‐gal treated cells (GEPE‐0).

### 3.3 GEP Improves Cognitive Impairment, Muscle Strength, and Dysfunction in Aging Mice

Based on the in vitro experimental results, we further verified the in vivo anti‐aging effect of GEP in a natural aging mouse model. In the 5‐day place navigation training of the MWM test, the escape latency of mice in all groups gradually decreased with prolonged training. Compared with the control group, aging mice exhibited significantly longer escape latency on each training day, suggesting impaired spatial learning acquisition. Medium‐ and high‐dose GEP intervention shortened the escape latency of aging mice, with the medium‐dose showing the most pronounced improvement (Figure 3B). In the spatial probe trial, aging mice spent significantly less time in the target quadrant and crossed the original platform position fewer times than the control group, indicating obvious spatial learning and memory impairment in aging mice, and GEP intervention significantly reversed these cognitive deficits (Figure 3C,D). Nest building test results showed that the nesting score of aging mice was significantly lower than that of the control group, indicating impaired social behavior and autonomous function, while GEP intervention significantly increased the nesting score and restored autonomous behavior in mice (Figure 3E). In addition, the limb grip strength and exercise endurance of aging mice were significantly lower than those of the control group, and GEP intervention effectively improved grip strength and endurance in mice, with the medium‐dose GEP (Aging_GEP_M) showing the best effect (Figure 3F,G).

**FIGURE 3:**
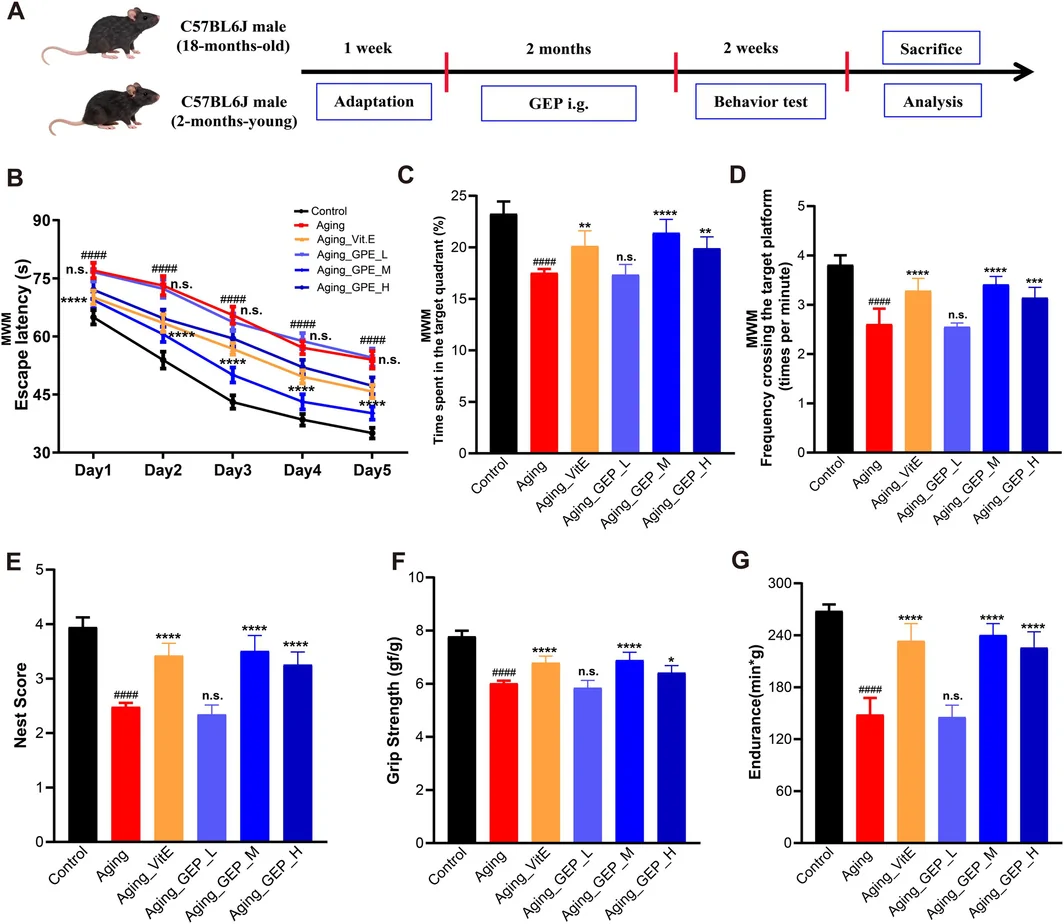
GEP improves cognitive deficit behaviors, muscle strength, and dysfunction in aging mice. (A) Schematic diagram of the experimental design. (B) Escape latency during 5‐day place navigation training. (C) The percentage of time spent in the target quadrant in the probe test. (D) The frequency across the target quadrant in the probe test. (E) The nest score in the nest building test. (F) Grip strength. (G) Endurance. All experimental data are presented as mean ± SD. *n* = 6, ns > 0.05, ^####^ *p* < 0.0001 versus Control group; *p < 0.05, **p < 0.01, ***p < 0.001, and ****p < 0.0001 versus Aging group.

### 3.4 GEP Improves Hippocampal Neuron Damage and Upregulates BDNF Expression in Aging Mice

To clarify the protective effect of GEP on brain aging, we further observed the histopathological changes in the hippocampus of mice. H&E staining showed that hippocampal neurons in the control group were neatly arranged, morphologically intact with clear nucleoli; in contrast, hippocampal neurons in the Aging group were disordered, with karyopyknosis and hyperchromatism, and the number of damaged neurons increased significantly; GEP intervention significantly alleviated these pathological changes and maintained the normal morphology and arrangement of neurons, with the medium‐dose GEP (Aging_GEP_M) showing the most significant protective effect (Figure 4A). BDNF is a core factor maintaining neuronal survival and synaptic function. IHC results showed that the positive expression level of BDNF in the hippocampus of aging mice was significantly lower than that in the control group; GEP intervention significantly upregulated BDNF expression in the hippocampus (Figure 4B). Notably, GEP exhibited an inverted U‐shaped dose–response pattern: the medium‐dose GEP group (Aging_GEP_M) showed the strongest protective effect, which was superior to that of the vitamin E positive control (Aging_VitE), while efficacy was slightly reduced at the high dose (Aging_GEP_H) (Figure 4A,B).

**FIGURE 4:**
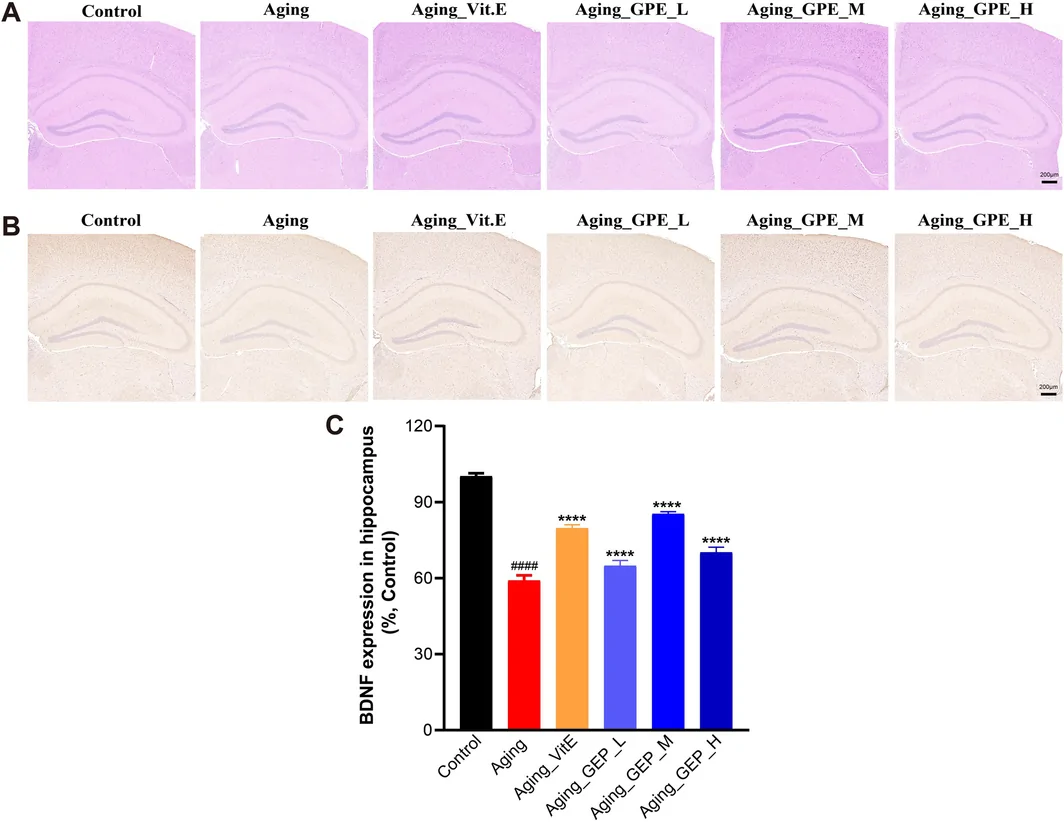
Effects of GEP on histopathological changes induced by hippocampal neuronal injury and BDNF levels in aging mice. (A) Representative micrographs of H&E staining of the hippocampal region in mouse brain tissues from different treatment groups. (B–C) The levels of BDNF in the hippocampal region of mouse brain tissues (B) and quantitative analysis (C). All experimental data are presented as mean ± SD. *n* = 6, ns > 0.05, ^####^ *p* < 0.0001 versus Control group; ****p < 0.0001 versus Aging group.

### 3.5 GEP Alleviates Oxidative Stress in Aging Mice via the Nrf2/Keap1 Signaling Pathway

Oxidative stress is one of the core pathological mechanisms of aging. We further detected oxidative stress indicators in the serum and brain tissue of mice. Compared with the control group, aging mice exhibited markedly elevated MDA levels and reduced SOD and CAT activities in both serum and brain tissue, indicating systemic and cerebral redox imbalance, while GEP treatment significantly reversed these oxidative stress abnormalities (Figure 5A–F). To further clarify the molecular mechanism, we detected the core proteins of the Nrf2/Keap1 antioxidant signaling pathway. The results showed that there was no significant difference in the protein level of Nrf2 in brain tissue among all groups, while the protein level of Keap1 in aging mice increased significantly, and the expression of antioxidant proteins HO‐1 and NQO1 decreased significantly; GEP intervention significantly downregulated Keap1 expression and significantly upregulated HO‐1 and NQO1 expression, activating the Nrf2/Keap1‐mediated antioxidant pathway, with medium‐dose GEP (Aging_GEP_M) showing the best effect (Figure 5G,H).

**FIGURE 5:**
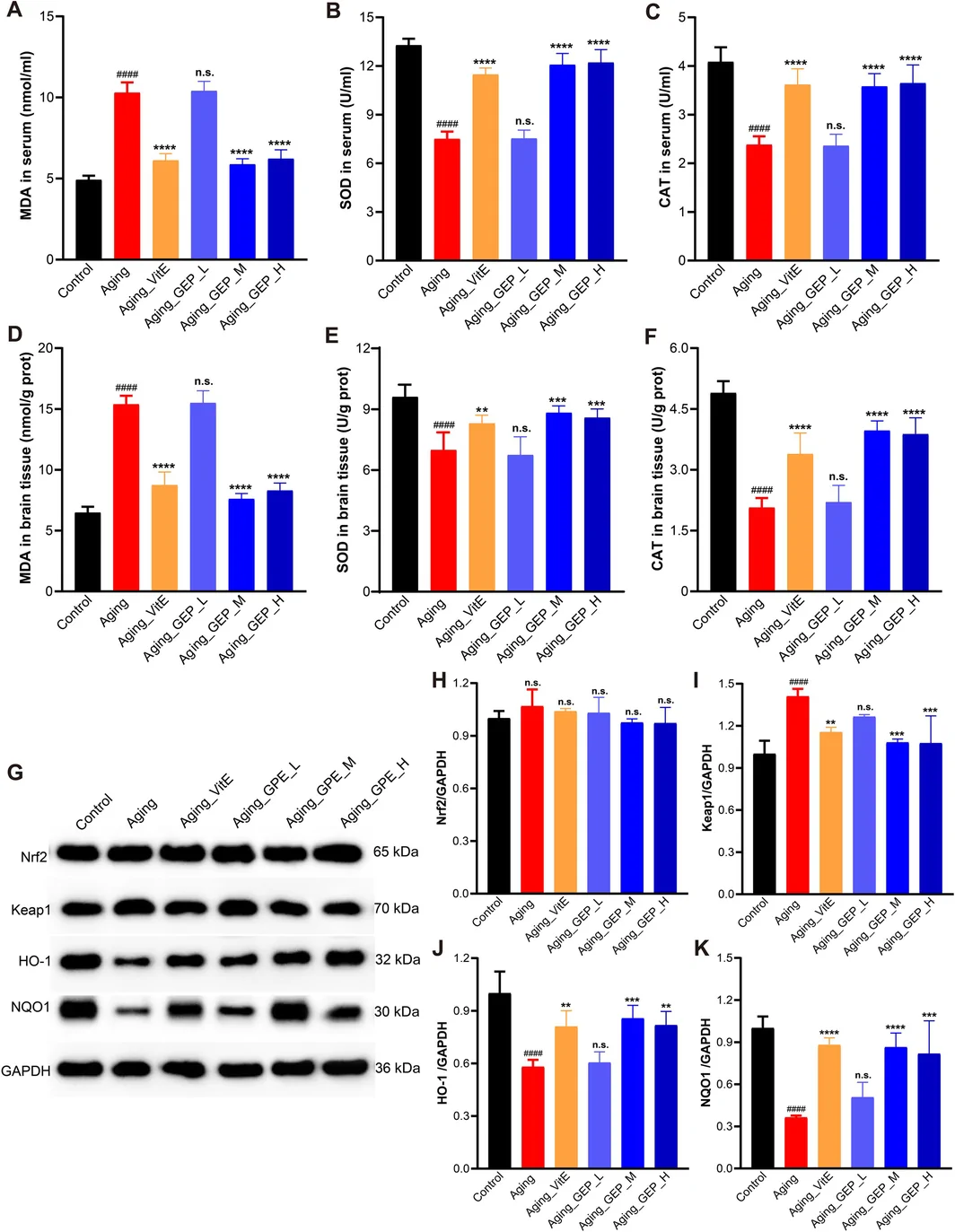
GEP reverses oxidative stress in aging mice via the Nrf2/Keap1 antioxidant signaling pathway. (A–C) Levels of MDA (A), SOD activity (B), and CAT activity (C) in mouse serum. (D–F) Levels of MDA (D), SOD activity (E), and CAT activity (F) in mouse brain tissues. (G, H) Representative protein bands of the Nrf2/Keap1 antioxidant signaling pathway in mouse brain tissues (G) and their quantitative analysis (H–K). All experimental data are presented as mean ± SD. *n* = 6, ns > 0.05, ^####^ *p* < 0.0001 versus Control group; **p < 0.01, ***p < 0.001, and ****p < 0.0001 versus Aging group.

### 3.6 GEP Alleviates Inflammatory Response in Aging Mice by Inhibiting the TLR4/NF‐κB Signaling Pathway

Aging is often accompanied by chronic low‐grade inflammation. We further detected the inflammatory level in each group of mice. ELISA results showed that the levels of pro‐inflammatory factors such as IL‐1β, IL‐6, and TNF‐α in the serum and brain tissue of aging mice were significantly higher than those in the control group, indicating that aging caused systemic and intracerebral inflammatory response; GEP intervention significantly reduced the levels of these pro‐inflammatory factors and effectively alleviated the inflammatory response (Figure 6A–F). To clarify the anti‐inflammatory mechanism, we detected the core proteins of the TLR4/NF‐κB signaling pathway. The results showed that the expression of TLR4 in the brain tissue of aging mice increased significantly, and the phosphorylation level of NF‐κB P65 was also significantly upregulated, indicating overactivation of the TLR4/NF‐κB pathway; GEP intervention significantly inhibited TLR4 expression and reduced the phosphorylation level of P65, thereby blocking the activation of the NF‐κB pathway and inhibiting the transcription and release of inflammatory factors, with medium‐dose GEP (Aging_GEP_M) showing the best effect (Figure 6G,H).

**FIGURE 6:**
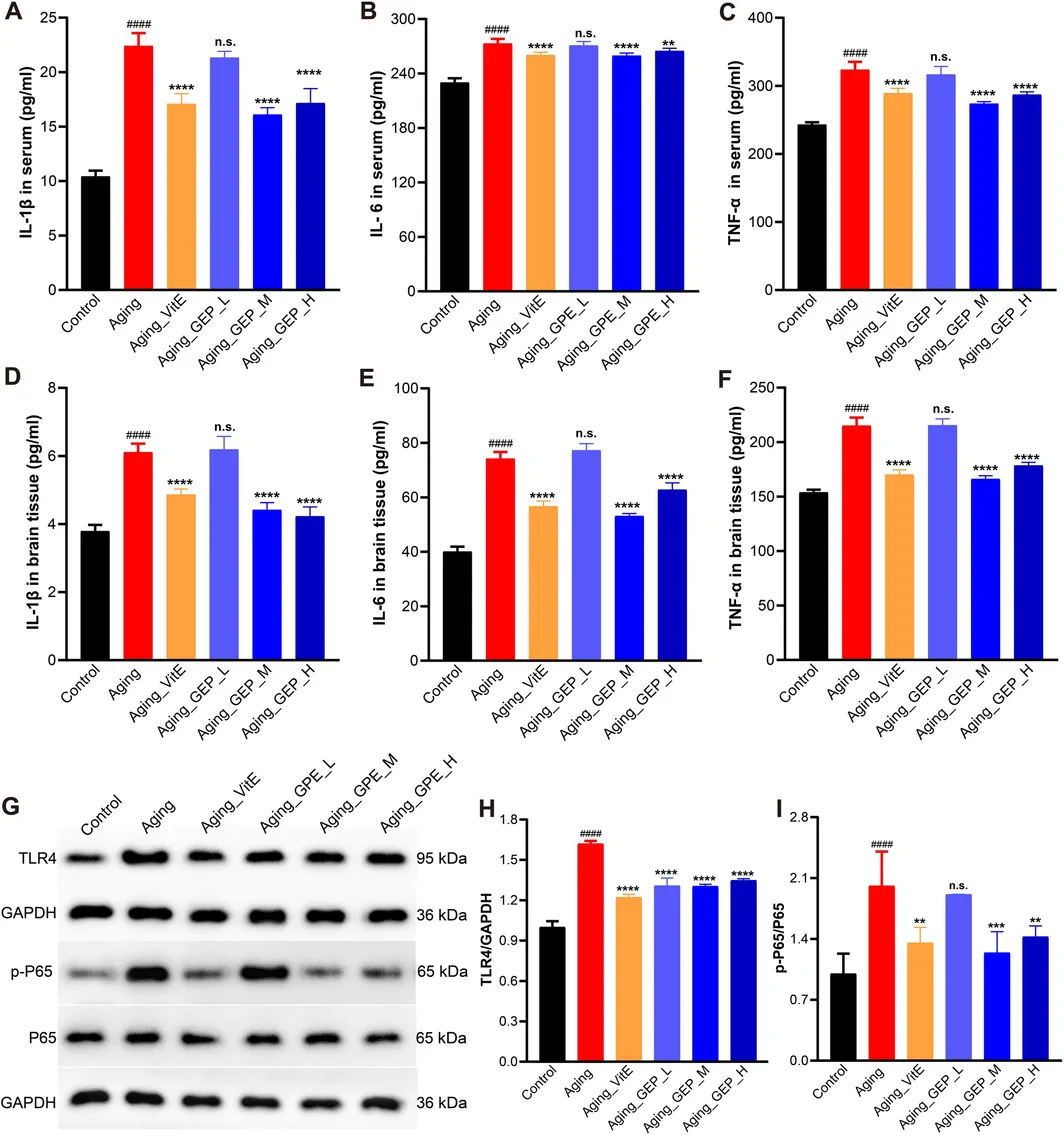
GEP alleviates inflammation in aging mice by inhibiting the TLR4/NF‐κB signaling pathway. (A–C) Levels of IL‐1β (A), IL‐6 (B), and TNF‐α (C) in mouse serum. (D–F) Levels of IL‐1β (D), IL‐6 (E), and TNF‐α (F) in mouse brain tissues. (G, H) Expression levels of representative proteins (TLR4 and P‐P65) of the TLR4/NF‐κB signaling pathway in mouse brain tissues (G) and their quantitative analysis (H, I). All experimental data are presented as mean ± SD. *n* = 6, ns > 0.05, ^####^ *p* < 0.0001 versus Control group; **p < 0.01, ***p < 0.001, and ****p < 0.0001 versus Aging group.

### 3.7 GEP Regulates Gut Microbiota Dysbiosis in Aging Mice

We analyzed the fecal microbiota of mice in each group using 16S rDNA gene sequencing technology. The results showed that the α‐diversity of aging mice decreased significantly, and GEP administration promoted its recovery to a certain extent (Figure 7A–D). Principal coordinate analysis (PCoA) confirmed that aging (Aging group) caused significant separation of mouse gut microbiota from the young control group (Control group), while after medium‐dose GEP (Aging_GEP_M) administration, samples in the Aging_GEP_M group gradually approached the control group, and the distance between the two groups was significantly shortened (Figure 7E). In addition, Venn diagram analysis showed that there were 114 common OTUs among the 6 groups, and the control group, Aging group, Aging_VitE group, Aging_GEP_L group, Aging_GEP_M group, and Aging_GEP_H group had 243, 84, 218, 182, 206, and 196 unique OTUs, respectively (Figure 7F). There were significant differences in the total number of OTUs among groups: the total number of OTUs in the Aging group was significantly lower than that in the control group, and after GEP administration, the total number of OTUs in aging mice was significantly restored, with medium‐dose GEP (Aging_GEP_M) showing the best effect (Figure 7F), which was consistent with the α‐diversity analysis results.

**FIGURE 7:**
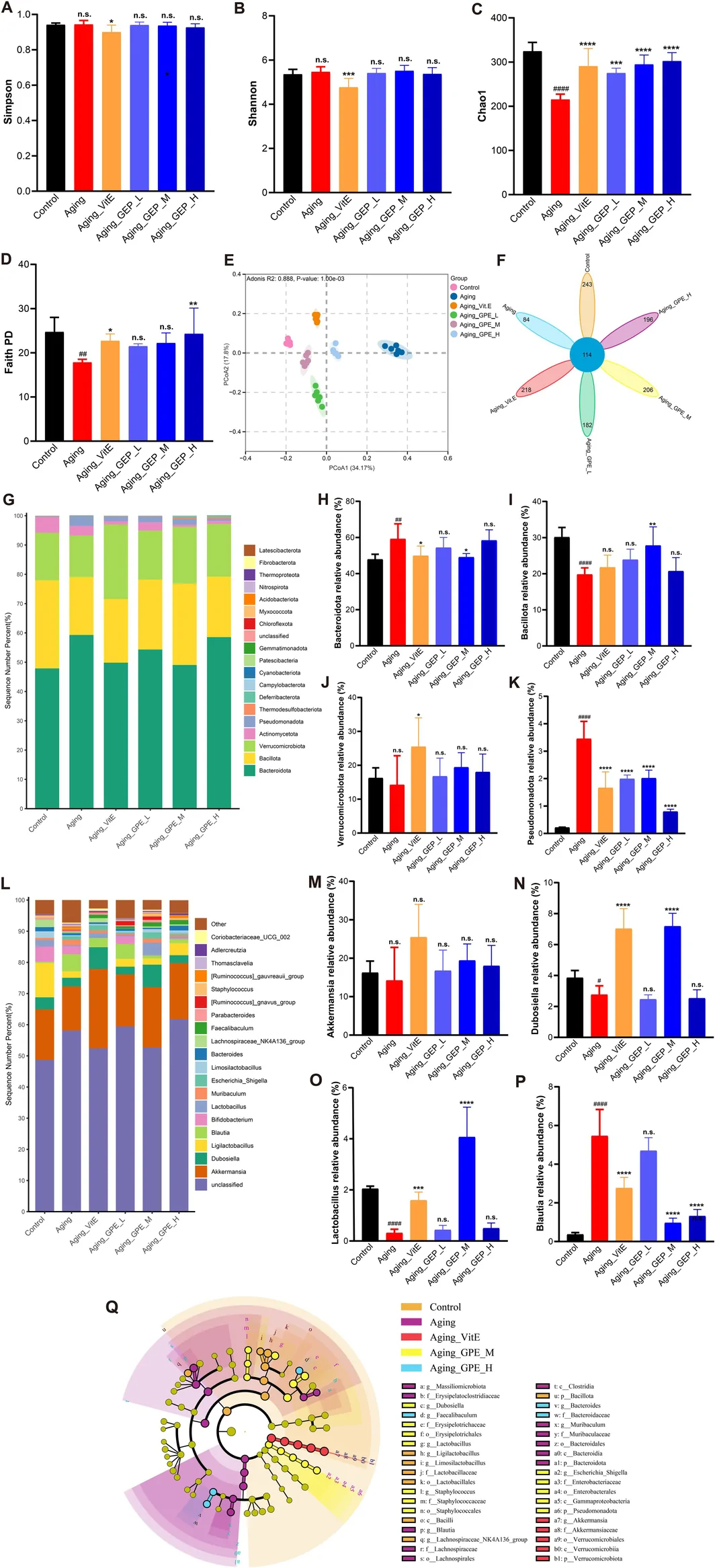
GEP regulates gut microbiota in aging mice. (A–D) α‐Diversity of bacterial communities in each group, including Simpson index (A), Shannon index (B), Chao1 index (C), and Faith index (D). (E) Principal co‐ordinates analysis (PCoA) of β‐diversity. (F) Venn diagram. (G) Relative abundance of gut microbiota at the phylum level. (H) Bacteroidota. (I) Bacillota. (J) Verrucomicrobiota. (K) Pseudomonadota. (L) Relative abundance of gut microbiota at the genus level. (M) *Akkermansia*. (N) *Dubosiella*. (O) *Lactobacillus*. (P) *Blautia*. (Q) LEfSe analysis of gut microbiota (LDA score > 4, cladogram showing differentially abundant taxa). All experimental data are presented as mean ± SD. *n* = 6, ns > 0.05, ^#^ *p* < 0.05, ^##^ *p* < 0.01, ^####^ *p* < 0.0001 versus Control group; *p < 0.05, **p < 0.01, ***p < 0.001, and ****p < 0.0001 versus Aging group.

Next, we further explored the regulatory effect of GEP on the composition of gut microbiota and related functional bacteria at the phylum and genus levels. At the phylum level, *Bacteroidota* was the dominant phylum in mouse fecal microbiota. Further analysis found that compared with the control group, the relative abundances of *Bacteroidota* and *Pseudomonadota* were significantly increased, while those of *Bacillota* and *Verrucomicrobiota* were notably and moderately decreased, respectively, in the feces of aging mice. GEP administration markedly reversed these abnormal changes, effectively reducing the abundances of *Bacteroidota* and *Pseudomonadota* and elevating the relative abundances of *Bacillota* and *Verrucomicrobiota* in aging mice. Among all treatments, the medium‐dose GEP group (Aging_GEP_M) exhibited the optimal overall intervention effect (Figure 7G–K). At the genus level, we further analyzed the abundance changes of core genera. The results showed that compared with the control group, the relative abundance of *Akkermansia* was slightly decreased, *Dubosiella* and *Lactobacillus* were significantly decreased, while *Blautia* was abnormally enriched in the feces of aging mice. GEP administration significantly increased the relative abundances of these beneficial genera (Figure 7L–O), indicating that GEP effectively reversed the decrease in beneficial bacteria abundance caused by aging. In addition, compared with the control group, the relative abundance of *Blautia* in the feces of aging mice increased abnormally and significantly, and GEP intervention significantly reversed the excessive enrichment of *Blautia* induced by aging (Figure 7P). Linear discriminant analysis effect size (LEfSe) analysis further showed that *Clostridia* (class), *Lachnospirales* (order), *Bacteroidales* (order), *Bacteroidia* (class), and *Bacteroidota* (phylum) were enriched in the Aging group; *Dubosiella* (genus), *Enterobacteriaceae* (family), *Pseudomonadota* (phylum), *Gammaproteobacteria* (class), and *Staphylococcus* (genus) were enriched in the Aging_GEP_M group; *Faecalibaculum* (genus), *Bacteroidaceae* (family), and *Bacteroides* (genus) were enriched in the Aging_GEP_H group (LDA score > 4) (Figure 7Q).

### 3.8 GEP Improves Intestinal Barrier Damage in Aging Mice

Intestinal barrier integrity is the core of maintaining intestinal homeostasis. We detected the expression of tight junction proteins in colon tissue by IF staining. The results showed that the fluorescence signals of ZO‐1 and Occludin in the colon tissue of Control mice were strong, indicating intact intestinal barrier structure; compared with the control group, the fluorescence intensities of ZO‐1 and Occludin in aging mice decreased significantly, indicating that aging caused the loss of tight junction proteins; GEP intervention significantly upregulated the expression of ZO‐1 and Occludin, with medium‐dose GEP (Aging_GEP_M) showing the best effect, and its ameliorative effect was stronger than that of vitamin E positive control (Aging_VitE) (Figure 8A–C), indicating that GEP has the potential to repair intestinal barrier damage in aging mice.

**FIGURE 8:**
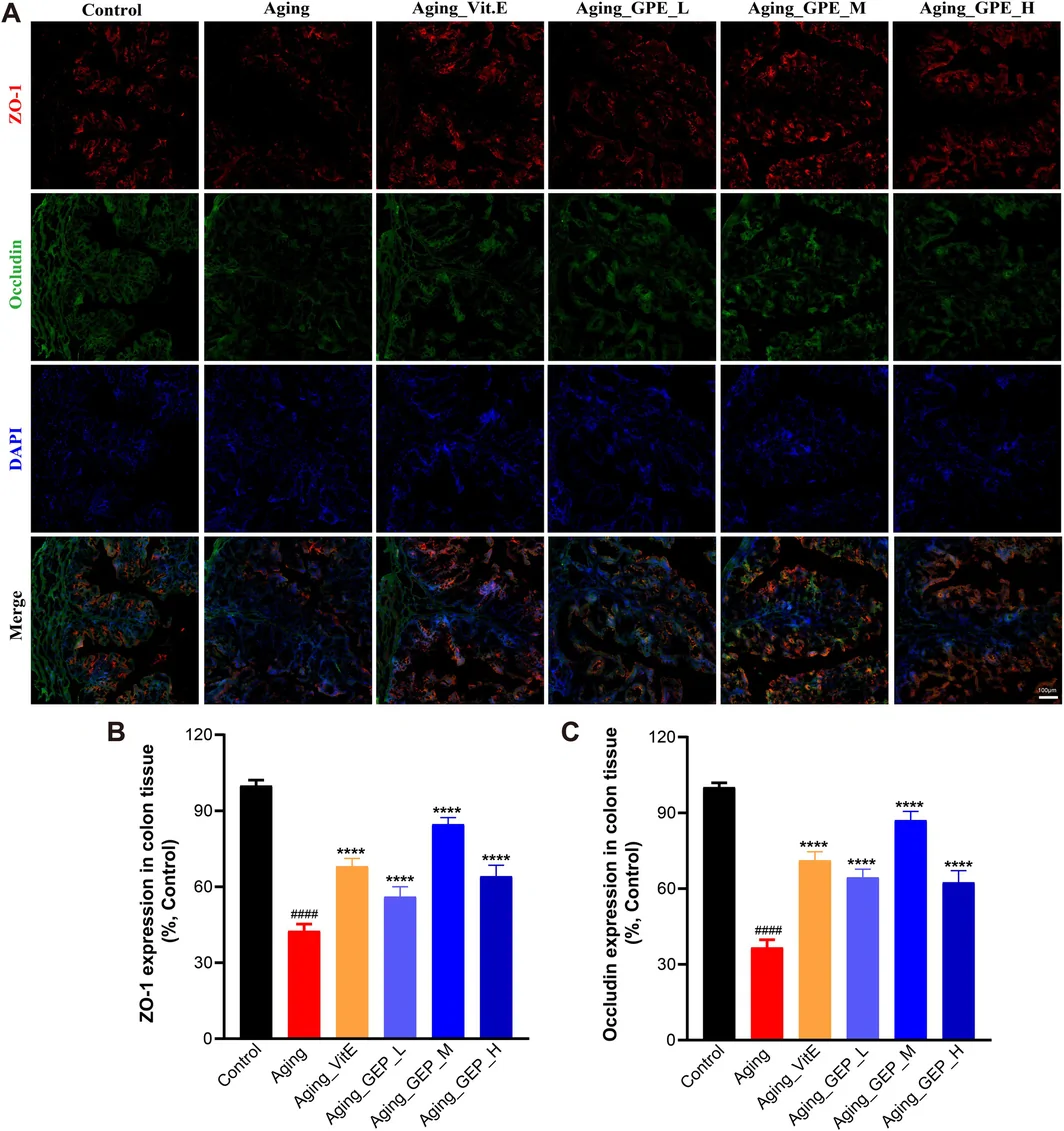
GEP ameliorates intestinal barrier damage in aging mice. (A–C) Expression levels of tight junction proteins ZO‐1 and Occludin in mouse colonic tissues (A) and quantitative analysis (B, C). All experimental data are presented as mean ± SD. *n* = 6, ns > 0.05, ^####^ *p* < 0.0001 versus Control group; ****p < 0.0001 versus Aging group.

## 4 Discussion

Numerous studies have reported that natural active ingredients can intervene in the aging process through multi‐dimensional regulation of oxidative stress, inflammatory response, and gut microbiota homeostasis, and alleviate aging‐related organ dysfunction (Fernandes et al. 2023; Teng et al. 2025; Li et al. 2026). In the present study, based on previous research, we found that GEP alleviated d‐gal‐induced microglial senescence in vitro and prolonged the lifespan of *C. elegans* ; in the natural aging mouse model, GEP effectively improved cognitive impairment and muscle dysfunction, alleviated hippocampal neuron damage, and upregulated BDNF expression. In addition, GEP exerted multi‐target anti‐aging effects by activating the Nrf2/Keap1 antioxidant pathway, inhibiting the TLR4/NF‐κB inflammatory signal, regulating gut microbiota structure, and repairing intestinal barrier damage. Therefore, this study mainly revealed that GEP systematically delays the natural aging process by synergistically regulating oxidative stress‐inflammation homeostasis‐gut microbiota, providing new experimental evidence for the development of natural polysaccharide anti‐aging substances.

Oxidative stress is one of the core driving factors of aging. Excessive accumulation of ROS during aging destroys the structure of cellular macromolecules, induces cellular senescence and apoptosis, and further aggravates tissue functional decline (Snieckute et al. 2023; Robinson et al. 2018; Yang et al. 2024). The Nrf2/Keap1 signaling pathway is the core pathway regulating the body's antioxidant defense. Under physiological conditions, Keap1 mediates the ubiquitination and degradation of Nrf2 to maintain the basal activity of the pathway; when oxidative stress occurs, Nrf2 dissociates and translocates into the nucleus, initiating the transcription of downstream antioxidant proteins such as HO‐1 and NQO1, eliminating excess ROS and restoring oxidative homeostasis (Ren et al. 2018; Rostagno and Ghiso 2025; Lu et al. 2026). Previous studies have confirmed that GEP alleviates oxidative damage in intestinal epithelial cells by activating the Nrf2 pathway (Yao et al. 2026), which is consistent with the results of this study. Our study found that the content of MDA in the serum and brain tissue of natural aging mice increased significantly, while the activities of antioxidant enzymes such as SOD and CAT decreased significantly, indicating that aging caused systemic and central oxidative imbalance. GEP intervention significantly reversed the abnormalities of these indicators, downregulated Keap1 expression, upregulated the protein levels of HO‐1 and NQO1, and activated the Nrf2‐mediated antioxidant defense system. Notably, Nrf2 protein detected in this study was total protein extracted from whole brain tissue. It is well established that Nrf2 activation is primarily mediated by its nuclear translocation from the cytosol, rather than a marked elevation in total cellular protein abundance, since Keap1 mainly regulates the ubiquitination and degradation of Nrf2 instead of its basal expression (Lv et al. 2023; Teng et al. 2025). The absence of significant change in total Nrf2 levels is therefore consistent with the canonical regulatory pattern of this pathway, and the robust upregulation of downstream antioxidant enzymes HO‐1 and NQO1 provides solid functional evidence for Nrf2 pathway activation by GEP. This indicates that GEP can delay the aging process of cells and tissues by enhancing the body's antioxidant capacity and eliminating oxidative damage products accumulated during aging. In addition to oxidative stress, chronic low‐grade inflammation (i.e., “inflammaging”) is also a key pathological feature of aging. Oxidative stress and inflammatory response form a mutually reinforcing vicious cycle, which jointly accelerates the progression of aging and related diseases (Matzkin et al. 2016; Chen, Ma, et al. 2025). The TLR4/NF‐κB signaling pathway is the core pathway regulating inflammatory response. Activation of TLR4 triggers the phosphorylation and nuclear translocation of downstream NF‐κB P65, initiating the transcription and release of pro‐inflammatory factors such as IL‐1β, IL‐6, and TNF‐α, and mediating tissue inflammatory damage (Kawai and Akira 2010; Wang et al. 2024; Ma, Zhu, et al. 2026). Previous studies have shown that GEP exerts anti‐inflammatory and neuroprotective effects by inhibiting the activation of the NF‐κB pathway (Gan et al. 2024). The results of this study showed that the levels of pro‐inflammatory factors in the serum and brain tissue of natural aging mice increased significantly, and the expression of TLR4 and the phosphorylation level of P65 were significantly upregulated, indicating that the inflammatory pathway in aging mice was overactivated; GEP intervention significantly inhibited the activation of the TLR4/NF‐κB pathway, reduced the release of pro‐inflammatory factors, and effectively alleviated systemic and central inflammatory responses. This suggests that GEP can alleviate aging‐related chronic inflammatory state by blocking the excessive activation of inflammatory signals and breaking the vicious cycle between oxidative stress and inflammation.

In recent years, an increasing number of studies have shown that gut microbiota plays a key regulatory role in the aging process. The hypothesis that “aging begins in the gut” has been confirmed by a large number of studies. Gut microbiota dysregulation can affect the function of the central nervous system through the gut–brain axis and mediate brain aging (Wilmanski et al. 2021; Bradley and Haran 2024; Cox et al. 2026). During aging, the diversity of gut microbiota decreases significantly, the abundance of beneficial bacteria decreases while that of harmful bacteria increases, and the integrity of the intestinal barrier is destroyed, leading to microbial translocation and endotoxemia, which further aggravate systemic inflammation and oxidative stress (Bana and Cabreiro 2019; Ticinesi et al. 2026; Jing et al. 2025). Previous studies have found that GEP can regulate the composition of gut microbiota and improve intestinal homeostasis (Xu et al. 2023; Gan et al. 2024), which is consistent with the findings of this study. Our results showed that the α‐diversity of gut microbiota in natural aging mice decreased significantly, and the microbial structure was significantly separated from that of young mice. The abundances of *Bacillota* and *Verrucomicrobiota* decreased, while those of *Bacteroidota* and *Pseudomonadota* increased in the feces of aging mice. Meanwhile, the abundances of beneficial bacteria such as *Dubosiella* and *Lactobacillus* decreased significantly, *Akkermansia* decreased slightly, and *Blautia* was abnormally enriched in the feces of aging mice. GEP intervention effectively reversed these abnormal changes of microbiota and restored the diversity and homeostasis of gut microbiota. Consistently, IF results confirmed that GEP markedly upregulated the colonic tight junction proteins ZO‐1 and occludin, effectively repairing aging‐induced intestinal barrier disruption. This observation aligns closely with the well‐established “leaky gut” theory of aging. Aging‐induced damage to intestinal tight junctions impairs mucosal integrity and increases intestinal permeability (Wang and Yang 2024; Conway et al. 2025; Man et al. 2015; Salazar et al. 2023). This pathological “leaky gut” permits large quantities of lipopolysaccharide (LPS, gut‐derived endotoxin) to translocate from the intestinal lumen into peripheral blood circulation. Circulating LPS subsequently crosses the compromised blood–brain barrier (BBB), specifically activating the TLR4/NF‐κB inflammatory signaling pathway in central nervous system resident microglia, thereby exacerbating neuroinflammation, neuronal senescence, and cognitive deterioration (Hajare and Kulkarni 2025; Zhao et al. 2026; Kalyan et al. 2022). By restoring tight junction protein abundance, GEP effectively seals the damaged intestinal epithelial barrier, blocks LPS translocation into systemic circulation, and further suppresses the downstream TLR4‐mediated neuroinflammatory cascade. This barrier‐protective effect ultimately contributes to improved intestinal homeostasis and alleviated central aging damage, which is consistent with GEP's beneficial effects on cognitive function and hippocampal neuron integrity observed in this study.

It is noteworthy that the molecular weight of purified GEP reaches 314 kDa, an extremely large macromolecular polysaccharide. Existing pharmacological consensus confirms that polysaccharides of such high molecular weight cannot freely penetrate the intact BBB to directly act on hippocampal neurons or microglia (Makani et al. 2016). Accordingly, the neuroprotective and anti‐senescence effects observed in brain tissue cannot stem from the direct central action of GEP itself. Instead, GEP's primary target organ is the gastrointestinal tract. GEP reshapes aging‐disrupted gut microbiota and repairs damaged intestinal barrier function, which may sequentially reduce systemic oxidative stress and peripheral inflammatory burden, and these circulating systemic improvements indirectly alleviate central neuroinflammation and neuronal injury via the gut–brain axis. Although this study did not quantitatively analyze microbial metabolites such as short‐chain fatty acids (SCFAs), which are the core microbial metabolites derived from intestinal commensal bacteria in our study, multiple published studies have demonstrated that enriched beneficial taxa such as *Akkermansia* and *Lactobacillus* can produce abundant microbial metabolites including SCFAs to suppress systemic inflammation (Wang, Xie, et al. 2025; Xu et al. 2025; Cao et al. 2026; Li et al. 2022). We speculate that the shifts in gut community composition induced by GEP may alter the production of microbial metabolites including SCFAs to mediate remote brain protection.

With the in‐depth study of aging mechanisms, more and more evidence shows that aging is not a pathological process caused by a single factor, but a complex process regulated by multiple systems and pathways. Single‐target intervention is often difficult to achieve ideal anti‐aging effects (López‐Otín et al. 2023; Rosoff et al. 2023; Kroemer et al. 2025). Natural polysaccharides, due to their multi‐component and multi‐target characteristics, show unique advantages in the field of anti‐aging (Teng et al. 2025). This study found that GEP can simultaneously regulate oxidative stress, inflammatory response and gut microbiota homeostasis, and systematically delay the natural aging process through the synergistic effect of multiple pathways, which also reflects the advantages of multi‐target intervention of TCM active ingredients. Notably, the anti‐aging efficacy of GEP exhibited a consistent inverted U‐shaped dose–response pattern across behavioral, biochemical, and molecular endpoints, with the medium dose (200 mg/kg) consistently outperforming the high dose (400 mg/kg). This phenomenon is widely documented for natural polysaccharides (Wen et al. 2025; Teng et al. 2025). Excessively high concentrations of polysaccharides may trigger mild pro‐oxidant reactions in peripheral tissues and overstimulate intestinal innate immune signaling, which in turn elevates the secretion of pro‐inflammatory cytokines and partially counteracts the antioxidant and anti‐inflammatory benefits conferred by GEP. Mechanistically, high‐dose GEP may over‐activate pattern recognition receptors such as TLR4 on intestinal epithelial cells and immune cells, reversing the inhibitory effect of moderate GEP on the TLR4/NF‐κB inflammatory cascade and weakening its synergistic antioxidant effect on the Nrf2/Keap1 pathway, ultimately leading to attenuated protective efficacy at the highest dose, but still needs further validation. Moreover, after GEP regulates gut microbiota, the specific metabolites or signaling molecules that mediate the regulation of the gut–brain axis still need to be further explored in the future. Additionally, this study only evaluated the protective effect of GEP in 20‐month‐old middle‐aged to old mice; whether these findings can be generalized to advanced aging stages (e.g., 24 months and older) requires further validation in older cohorts. Another limitation is the lack of glycosidic linkage characterization for GEP. Despite α‐d‐glucan identity supported by monosaccharide profiling and FT‐IR, methylation and 2D NMR assays were not performed to identify 1,3‐, 1,4‐ and 1,6‐linkages. Linkage types govern microbial degradability and carbon utilization by taxa including *Akkermansia*, precluding an explicit structure‐fermentability correlation in this study. Future work will combine methylation, 2D NMR and in vitro fermentation to elucidate the linkage‐microbiota structure–activity relationship.

## 5 Conclusion

In conclusion, our study verified that GEP attenuated d‐gal‐induced senescence in BV2 microglia and prolonged the lifespan of *C. elegans* N2. In naturally aging mice, GEP markedly ameliorated cognitive and muscle dysfunction, alleviated hippocampal neuronal damage, and upregulated neuroprotective BDNF expression. Mechanistically, GEP exerted anti‐aging effects by activating the Nrf2/Keap1 pathway to mitigate oxidative stress, inhibiting the TLR4/NF‐κB pathway to suppress chronic inflammation, regulating aging‐related gut microbiota dysbiosis, and upregulating ZO‐1/Occludin to repair intestinal barrier damage. Collectively, these findings demonstrate that GEP delays natural aging via the synergistic regulation of oxidative stress, inflammatory responses, and gut microbiota–intestinal barrier homeostasis, supporting its development as a functional ingredient for aging intervention.

## Author Contributions

**Huihuang Shi:** conceptualization, methodology, data curation, formal analysis, investigation, writing – original draft, validation. **Shunqiang Yang:** conceptualization, methodology, formal analysis, data curation, writing – original draft, project administration. **Yue Chen:** data curation, investigation, supervision, software, writing – original draft. **Biting Shaog:** visualization, supervision, writing – original draft, validation. **Rui Gong:** visualization, writing – original draft, validation. **Zhu Li:** conceptualization, methodology, data curation, formal analysis, investigation, visualization, writing – original draft, writing – review and editing, project administration, funding acquisition.

## Funding

This study was supported by the Yunnan Fundamental Research Projects (No. 202501AU070176), the Open Project Program of Yunnan Key Laboratory of Gastrodia and Fungi Symbiotic Biology (No. TMKF2025B08), the Project of Scientific Research Start‐up Funds for Doctoral Talents of Zhaotong University—Zhu Li (No. 20240003), the Scientific and Technological Innovation Team Project under the Xingzhao Talent Support Program of Zhaotong City (No. F212250055), the Undergraduate Education and Teaching Reform Research Project of Zhaotong University (No. Ztjx202608), and the Open Project Program of Yunnan Key Laboratory of Smart Villages and Agri‐Cultural‐Tourism Integration.

## Disclosure

*AI Use Statement*: The author(s) declared that generative AI was not used in the creation of this manuscript.

## Conflicts of Interest

The authors declare no conflicts of interest.

## Data Availability

The data supporting the findings of this study are available from the corresponding author upon reasonable request. Data deposition: 16S rRNA sequencing raw data have been deposited to the National Center for Biotechnology Information (NCBI) under the BioProject number PRJNA1515948 (https://www.ncbi.nlm.nih.gov/bioproject/PRJNA1515948).
